# Bone marrow pericyte dysfunction in individuals with type 2 diabetes

**DOI:** 10.1007/s00125-019-4865-6

**Published:** 2019-04-17

**Authors:** Giuseppe Mangialardi, David Ferland-McCollough, Davide Maselli, Marianna Santopaolo, Andrea Cordaro, Gaia Spinetti, Maria Sambataro, Niall Sullivan, Ashley Blom, Paolo Madeddu

**Affiliations:** 1 Bristol Heart Institute, University of Bristol, Bristol Royal Infirmary, Level 7, Upper Maudlin Street, Bristol, BS2 8HW UK; 2 IRCCS Multimedica, Milan, Italy; 3 Department of Biochemistry, University of Sassari, Sassari, Italy; 4 Department of Specialized Medicines, Endocrine, Metabolic and Nutrition Diseases Unit, Santa Maria of Ca’ Foncello Hospital, Treviso, Italy; 5 Muscloskeletal Research Unit, School of Clinical Sciences, University of Bristol, Bristol, UK

**Keywords:** Angiocrine factors, Bone marrow, Diabetes, Microangiopathy, Pericytes

## Abstract

**Aims/hypothesis:**

Previous studies have shown that diabetes mellitus destabilises the integrity of the microvasculature in different organs by damaging the interaction between pericytes and endothelial cells. In bone marrow, pericytes exert trophic functions on endothelial cells and haematopoietic cells through paracrine mechanisms. However, whether bone marrow pericytes are a target of diabetes-induced damage remains unknown. Here, we investigated whether type 2 diabetes can affect the abundance and function of bone marrow pericytes.

**Methods:**

We conducted an observational clinical study comparing the abundance and molecular/functional characteristics of CD146^+^ pericytes isolated from the bone marrow of 25 individuals without diabetes and 14 individuals with uncomplicated type 2 diabetes, referring to our Musculoskeletal Research Unit for hip reconstructive surgery.

**Results:**

Immunohistochemistry revealed that diabetes causes capillary rarefaction and compression of arteriole size in bone marrow, without changing CD146^+^ pericyte counts. These data were confirmed by flow cytometry on freshly isolated bone marrow cells. We then performed an extensive functional and molecular characterisation of immunosorted CD146^+^ pericytes. Type 2 diabetes caused a reduction in pericyte proliferation, viability, migration and capacity to support in vitro angiogenesis, while inducing apoptosis. AKT is a key regulator of the above functions and its phosphorylation state is reportedly reduced in the bone marrow endothelium of individuals with diabetes. Surprisingly, we could not find a difference in AKT phosphorylation (at either Ser473 or Thr308) in bone marrow pericytes from individuals with and without diabetes. Nonetheless, the angiocrine signalling reportedly associated with AKT was found to be significantly downregulated, with lower levels of fibroblast growth factor-2 (FGF2) and C-X-C motif chemokine ligand 12 (CXCL12), and activation of the angiogenesis inhibitor angiopoietin 2 (ANGPT2). Transfection with the adenoviral vector carrying the coding sequence for constitutively active myristoylated AKT rescued functional defects and angiocrine signalling in bone marrow pericytes from diabetic individuals. Furthermore, an ANGPT2 blocking antibody restored the capacity of pericytes to promote endothelial networking.

**Conclusions/interpretation:**

This is the first demonstration of pericyte dysfunction in bone marrow of people with type 2 diabetes. An altered angiocrine signalling from pericytes may participate in bone marrow microvascular remodelling in individuals with diabetes.

**Electronic supplementary material:**

The online version of this article (10.1007/s00125-019-4865-6) contains peer-reviewed but unedited supplementary material, which is available to authorised users.

## Introduction

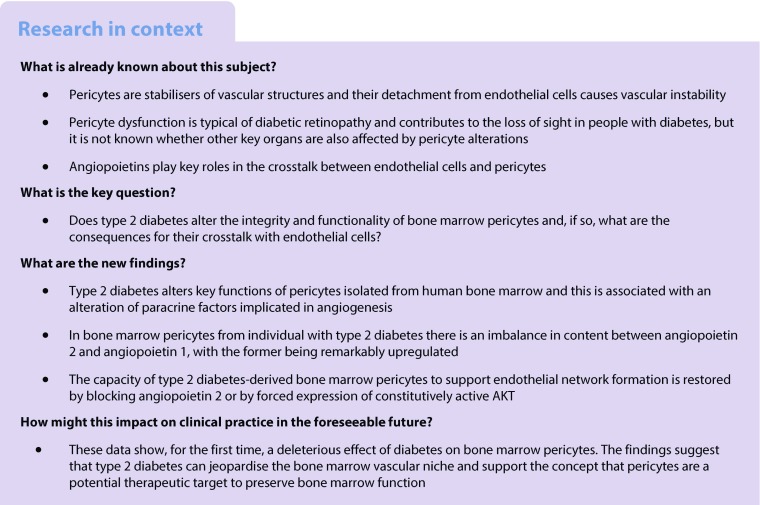



Diabetes mellitus represents an international health burden [1, 2], with cardiovascular disease remaining the most prevalent cause of morbidity and mortality [3].

Pathological phenomena affecting the bone marrow contribute to cardiovascular complications in people with diabetes [4–7]. Fadini and colleagues demonstrated that levels of circulating bone marrow-derived proangiogenic cells inversely correlate with complications in the coronary, peripheral and cerebrovascular regions [8]. Moreover, reduced counts of circulating CD34^+^ haematopoietic stem/progenitor cells (HSPCs) predict mortality in diabetic individuals [9], an association confirmed by a meta-analysis of 21 studies [10].

Reciprocally, the bone marrow represents a previously unforeseen target of diabetes-induced microvascular damage. A specific form of microangiopathy occurs in the bone marrow of diabetic mice [11]. In individuals with type 2 diabetes, bone marrow microangiopathy is associated with fat accumulation and apoptotic reduction of CD34^+^ haematopoietic cells [12–14]. Oxidative stress plays a pivotal role in the pathogenesis of bone marrow microangiopathy through the reduction of AKT phosphorylation/activity in endothelial cells [15, 16]. There are reports indicating pericyte loss and microaneurysm formation in the retina, kidney and brain of diabetic individuals [17, 18]. However, no previous investigation has determined the impact of type 2 diabetes on pericytes in human bone marrow.

Distinct pericyte-like cells have been described in the vascular niche of the murine bone marrow [19–24]. Vascular sinusoids are surrounded by leptin receptor (LEPROT)–platelet-derived growth factor receptor α (PDGFRα)-expressing cells [22, 25] and chemokine (C-X-C motif) ligand 12 (CXCL12)-abundant reticular cells [24]. An additional subtype reportedly resides in the proximity of bone marrow arterioles, is surrounded by neuronal fibres and expresses neural/glial antigen 2 (NG2) and nestin [26]. Two independent groups reported that human bone marrow contains clonogenic pericytes, which express the adhesion molecule CD146 together with the mesenchymal/pericyte markers α-smooth muscle actin (αSMA), NG2 and platelet-derived growth factor receptor β (PDGFRβ) but are negative for the haematopoietic and endothelial markers CD45, CD34 and CD31 [27, 28]. Bone marrow pericytes play key roles in the control of vascular tone and permeability and participate in the regulation of haematopoiesis through a molecular signalling involving CXCL12, stem cell factor–KIT ligand (KITLG) and angiopoietin 1 (ANGPT1) [29–32]. In vitro, bone marrow pericytes show tri-lineage mesenchymal differentiation potential and, upon transplantation into immunodeficient mice, they reportedly promote the formation of heterotopic haematopoietic niches through paracrine mechanisms [23, 28, 33].

This translational study aimed to compare the abundance and molecular/functional diversity of bone marrow pericytes from individuals with or without type 2 diabetes.

## Methods

### Ethics

Patients undergoing hip replacement surgery were recruited to the study, under informed written consent, at the Avon Orthopaedic Centre, Southmead Hospital, Bristol, UK. The study protocol complied with principles of the Declaration of Helsinki, was covered by institutional ethical approval (REC14/SW/1083 and REC14/WA/1005) and was registered as an observational clinical study in the NIHR Clinical Research Network Portfolio, UK Clinical Trials Gateway.

### Inclusion and exclusion criteria

The main clinical characteristics of the participants are included in Table 1. Type 2 diabetes was diagnosed according to the American Diabetes Association guidelines and was defined as follows: (1) patient/referring doctor reports a previous diagnosis of diabetes; (2) HbA_1c_ >48 mmol/mol (9.3%) and (3) off insulin for at least 12 months after diagnosis. Exclusion criteria comprised acute disease/infection, immune diseases, haematological disorders or malignancy, unstable angina, recent (within 6 months) myocardial infarction or stroke, critical limb ischaemia, liver failure, renal failure, pregnancy and lack of consent to participate. Randomisation was not required in the experimental plan. At all the occasions, a single patient’s sample was delivered from the Orthopaedic Centre to our laboratory and freshly processed according to the described protocols. Experimenters were aware the sample was from a diabetic or non-diabetic individual. Measurements were taken from distinct samples and each sample was generally measured in duplicate or triplicate, unless specified differently.

**Table 1:** Characteristics of participants

Characteristic	Without diabetes	With diabetes
Participants, *n*	25	14
Age, years	65 ± 2 (39–84)	67 ± 3 (41–79)
Men/women, *n*	11/14	7/7
BMI, kg/m^2^	29 ± 1 (21–42)	33 ± 2 (24–44)
HbA_1c_, mmol/mol	40 ± 1	60 ± 4
HbA_1c_, %	8.6	10.4

Data are mean±SD (range) or mean±SD, unless stated otherwise

### Tissue processing

The femoral head was removed as a standard step of hip replacement surgery. Bone marrow was scooped into a sterile pot, transferred into Falcon tubes containing PBS-EDTA (ThermoFisher, Gloucester, UK, catalogue number 28348) and placed in a fridge for collection within 1 h. Only material otherwise discarded was collected for the study.

### Cell isolation and expansion

Bone marrow samples were stratified on Ficoll Histopaque 1077 (Sigma-Aldrich, St. Louis, MO, USA, catalogue number 10771) and centrifuged at 300 *g* for 30 min at 25°C. Mononuclear cells sedimented at the interphase were then collected, washed twice with PBS and assessed for viability by trypan blue staining (ThermoFisher, catalogue number 15250061). An average of 1 × 10^8^ bone marrow mononuclear cells (BM-MNCs) was labelled with CD34-conjugated microbeads (Miltenyi, Woking, UK) and immunomagnetically sorted. CD34-depleted cells were labelled with CD45-conjugated microbeads (Miltenyi) and further sorted. The CD34–CD45 double-negative population was labelled with CD146-conjugated microbeads (Miltenyi) and enriched through immunomagnetic sorting. The purity of the selected cell population was assessed by flow cytometry (see below). Samples with a purity below 90% were excluded from the study.

The CD34^−^CD45^−^CD146^+^ cell fraction was then seeded onto 24-well plates at a density of 1 × 10^3^ to 5 × 10^3^ cells per cm^2^ and expanded in an α-MEM basal media (ThermoFisher Scientific, catalogue number 32561-029) supplemented with 20% FBS (ThermoFisher Scientific, catalogue number 16000044). Four to six cell lines per group were studied between passage three and seven in the subsequent experiments.

### Flow cytometry

Bone marrow mononuclear cells were labelled with primary antibodies (ESM Table 1) in staining buffer (PBS supplemented with 1% bovine serum albumin, Sigma, catalogue number A2058) for 30 min at 4°C, washed with cold PBS and resuspended in staining buffer. They were then acquired using a FACScantoII (BD Biosciences, Wokingham, UK). Quantification was performed using the FlowJo v10 software (FlowJo, Ashland, OR, USA). Flow cytometry antibodies used are reported in ESM Table 1.

### Western blot analyses

Protein extracts (20 μg) were separated by SDS-PAGE, transferred to PVDF membranes (Amersham-Pharmacia) and then probed with the antibodies listed in ESM Table 2.

### Immunohistochemistry

Portions of bone marrow were fixed in formalin 37% for 16 h, decalcified in 20% EDTA – 2% HCl solution for 4 h and then embedded in paraffin. The samples were sectioned on a rotary microtome at 2 μm, dried, deparaffinised and rehydrated. Antigen retrieval was performed by boiling the samples in a citrate buffer (10 mmol/l, Sigma, catalogue number P4809) at pH 6. After blocking non-specific binding with non-immune goat serum (ThemoFisher, catalogue number 10000C), sections were washed and then incubated with the following primary antibodies indicated in ESM Table 3: polyclonal mouse anti-human melanoma cell adhesion molecule (MCAM, BD Biosciences), polyclonal rabbit anti-von Willebrand factor (VWF, Abcam, Cambridge, UK), rabbit monoclonal anti-CD146 (Abcam), mouse monoclonal anti-protein gene product 9.5 (PGP9.5, Abcam), or monoclonal mouse anti-αSMA (Dako, Ely, UK) in PBS. All incubations were performed overnight at 4°C. The proper secondary antibodies (ESM Table 3), goat anti-rabbit or anti-mouse IgG (Alexa Fluor labelled), diluted 1:200 in PBS, were incubated for 60 min at 37°C. Nuclei were counterstained with 4′,6-diamidino-2-phenylindole (DAPI, 1 μg/μl, ThermoFisher, Catalogue number D1306). To remove the excess of autofluorescence, the bone marrow sections were immersed in a solution of 0.1% wt/vol. Sudan black (Sigma-Aldrich, catalogue number 86015) in 70% ethanol for 10 min at room temperature.

### Immunocytochemistry

Cells were seeded in eight-chamber slides, grown until confluence and then fixed with 4% wt/vol. paraformaldehyde for 15 min at room temperature. This step was followed by an incubation overnight at 4°C with the primary antibodies reported in ESM Table 3: rabbit anti-human CD146 (Abcam), rabbit anti-human CXCL12 (Cell Technologies, Cambridge, UK), rabbit anti-human vasclular endothelial-cadherin (VE-cadherin, Abcam), mouse anti-human nestin (Abcam), rabbit anti-human PDGFRβ (Santa Cruz, Heidelberg, Germany), rabbit-anti-human vascular endothelial growth factor receptor 2 (VEGFR2, Abcam), rabbit LEPROT (Abcam), and mouse VWF (Abcam). All incubations were performed overnight at 4°C in PBS. Then, the cells were washed with PBS and incubated with appropriate secondary antibody diluted 1:200 in PBS for 2 h at room temperature. Finally, cells were washed with PBS and counterstained with DAPI 1 μg/μl, for 1 min at room temperature. Images of random fields were obtained at ×200 magnification. Antibodies were validated previously by using negative and/or positive cell cultures as controls, according to the literature. Negative controls have been performed for each immunohistochemistry and immunocytochemistry experiment omitting the primary antibody.

### Viral infection

Bone marrow CD146^+^ cells were infected with an adenoviral vector carrying the coding sequence for constitutively active myristoylated AKT (Ad-myr*AKT*) or the empty vector Ad66 (Ad-Null) as a control (both from Addgene, Watertown, MA, USA). Cells were grown in complete medium in six-well plates until reaching 60–70% confluence and then infected for 24 h with adenoviral vectors at 100 multiplicity of infection. The medium was then changed and cells were used for experiments after 48 h. Effective expression of the transgene was confirmed by an activity assay, as previously reported [15].

### Functional assays

All the assays were performed with cells maintained in 5 mmol/l glucose.

#### Viability

A colorimetric method (MTS assay; Promega, Southampton, UK) was used for determining the number of viable cells. A total of 3 × 10^4^ cells was seeded into 96-well plates in quadruplicate. The absorbance was measured at λ 492 nm using a microplate reader (Multiskan Ascent; ThermoFisher Scientific).

#### Proliferation

The bromodeoxyuridine/5-bromo-2′-deoxyuridine (BrdU, Sigma-Aldrich) colourimetric assay was used to measure proliferation. The absorbance of the samples was measured in a microplate reader at 370 nm.

#### Apoptosis

Confluent cells were trypsinised and counted with trypan blue. Cells were washed twice with cold PBS and resuspended in 1X Annexin V Buffer (ThermoFisher Scientific) at 1 × 10^6^ cells/ml. Following this, 5 μl of FITC-conjugated Annexin V (BD Biosciences) was added to 100 μl of the cell suspension. Samples were acquired on a FACSCanto II flow cytometer and 1:400 wt/vol. propidium iodide staining solution (Invitrogen, Waltham, MA, USA) was added before the acquisition. Annexin V-positive/propidium iodide-negative cells were considered to be apoptotic. Results were analysed using FlowJo v10.

#### Migration

Cells were seeded on the upper part of 24-transwell plate filters pre-coated with fibronectin. The lower wells contained basal medium supplemented with recombinant PDGFB (100 ng/ml; Peprotech, London, UK) as chemoattractant. After overnight incubation, five random fields of the filter were counted at magnification ×200 per each filter using an Olympus BX40 microscope (Southend-on-Sea, UK).

#### In vitro network formation on Matrigel

HUVECs were purchased from Lonza (catalogue number C2519A) and checked for exclusion of mycoplasma infection. Expanded CD34^−^CD45^−^CD146^+^ cells (1 × 10^4^) and human umbilical vein endothelial cells (HUVECs, 2 × 10^4^) or HUVECs alone (3 × 10^4^) were suspended in a total volume of 100 μl α-minimal essential medium (αMEM) or in 50 μl of αMEM plus 50 μl of conditioned medium obtained from expanded CD146^+^ cells. The suspension was then added on top of 100 μl gelified, growth-enriched Matrigel (BD Biosciences) in each well of 96-well plate. Images were obtained using an inverted microscope (Zeiss, Birmingham, UK) at magnification ×200. In selected experiments, the assay was conducted in the presence of a blocking angiopoietin 2 (ANGPT2) antibody (1 μg/ml; ThermoFisher, catalogue number, PA1-20178) or goat IgG isotype control (1 μg/ml; Thermo Fisher, catalogue 02-6202).

### Gene expression analysis

#### RNA extraction and purification

RNA was extracted from immunosorted bone marrow pericytes following the QIAzol protocol and resuspended in 200 μl RNase-free H_2_O (DEPC-treated; ThermoFisher). Further purification was achieved using an acid-phenol/chloroform phase separation method. A NanoDrop 1000 spectrophotometer (Thermo Scientific) was used to quantify RNA and assess purity by 260/280 nm and 260/230 nm ratios.

#### cDNA reverse transcription

The High-Capacity RNA-to-cDNA Kit (Applied Biosystems, Warrington, UK) was used for complementary DNA (cDNA) synthesis.

#### Quantitative PCR

Quantitative PCR (qPCR) was used to measure the gene expression in CD146 immunosorted pericytes. Primers are shown in ESM Table 4 (all from Sigma-Aldrich, Gillingham, UK). The experiment was done using three to six biological samples per experimental group. The qPCR analysis was performed using relative quantification. The fold-differences between cells from participants with and without diabetes were evaluated by the Livak method (${2}^{-\varDelta \varDelta {\mathrm{C}}_{\mathrm{t}}}$). *GAPDH* and 18s rRNA were used as housekeeping genes.

### ELISA on conditioned media

Fibroblast growth factor 2 (FGF2), vascular endothelial growth factor (VEGF) A and B, CXCL12, ANGPT1, ANGPT2, IGFBP2 and SEMA6 were quantified using individual cytokine-specific ELISA kits (R&D Systems, Abingdon, UK).

### Statistical analysis

A Kolmogorov–Smirnov test was used to assess whether the data had a parametric distribution. Values are presented as mean ± SEM. Two-tailed independent samples *t* test was used to compare two groups assuming equal variances. ANOVA was used to compare multiple groups experiments, followed by Bonferroni post hoc *t* test to compare each group individually. All analyses were carried out using GraphPad Prism 7.0 (San Diego, CA, USA). A *p* value of <0.05 was considered statistically significant.

## Results

### Immunohistochemistry of pericytes in human bone marrow

**Fig. 1:**
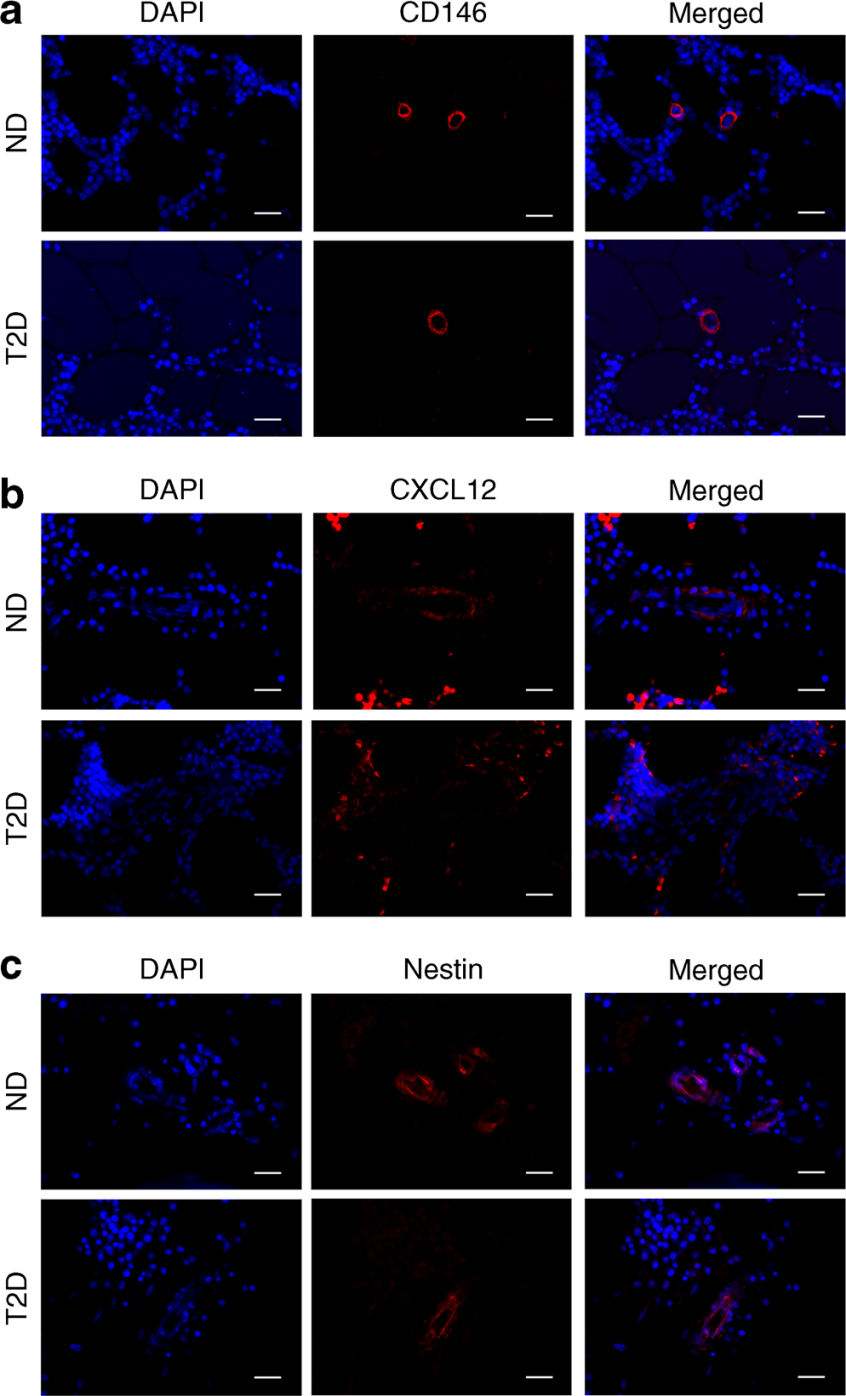
Characterisation of bone marrow pericytes in situ. Representative fluorescence microscopy images showing expression of CD146 (**a**), CXCL12 (**b**) and nestin (**c**) by cells localised around bone marrow capillaries of non-diabetic and diabetic individuals. Scale bars, 20 μm. ND, non-diabetic; T2D, type 2 diabetes

By immunofluorescence microscopy, we verified that pericytes expressing CD146, CXCL12, nestin and αSMA localise around capillaries and in the proximity of PGP9.5-positive neuronal fibres in the bone marrow of diabetic and non-diabetic individuals (Fig. 1a–c).

**Fig. 2:**
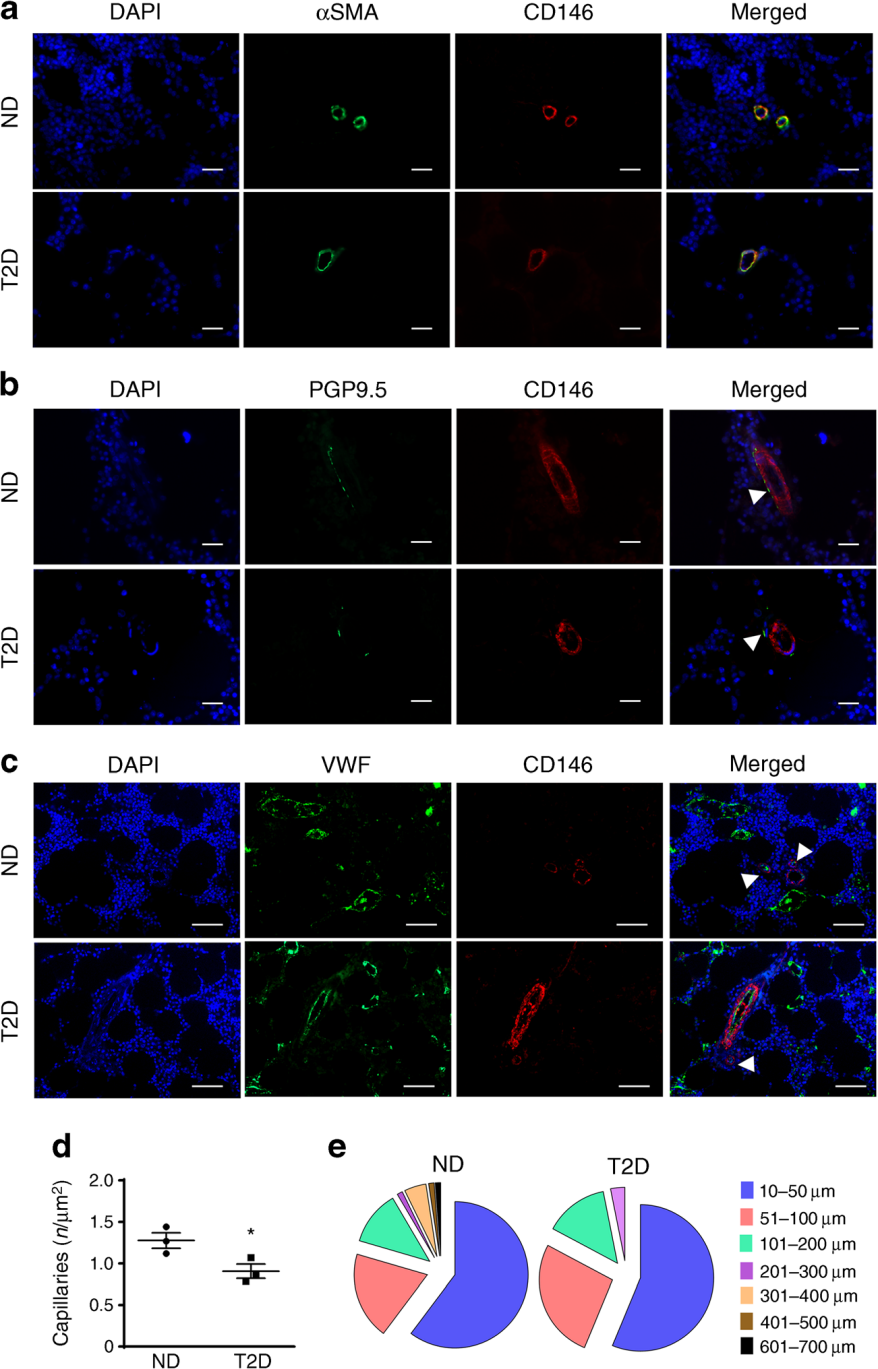
Immunohistochemical characterisation of CD146^+^ pericytes in human bone marrow. (**a**, **b**) Representative fluorescence microscopy images showing that CD146^+^ cells (red) express αSMA (green) (**a**) and co-localise with nerve profiles expressing PGP9.5 (green) (**b**) in capillaries of bone marrow from both non-diabetic and diabetic individuals. Scale bars, 20 μm. (**c**) Immunohistochemistry analysis of bone marrow vasculature performed by staining for CD146 (red) and VWF (green). Scale bars, 50 μm. Arrows point to PGP9.5- and VWF-positive structures. (**d**) Quantification of VWF-positive capillaries. Data are expressed as individual values and mean ± SEM; *n*=3 per group. *p<0.05, Student’s *t* test. (**e**) Distribution of arterioles according to their size. ND, non-diabetic; T2D, type 2 diabetes

Diabetes has profound remodelling effects on the bone marrow microvasculature [11, 34]. However, the impact on pericytes has not been previously investigated. We stained bone marrow samples using CD146 and VWF antibodies to distinguish endothelial cells (positive for both the antigens) from pericytes (expressing CD146 only) (Fig. 2c). As previously described [11, 12, 34], we observed a decrease in VWF-positive capillaries in the bone marrow of the diabetic compared with non-diabetic individuals (0.9 ± 0.15 vs1.27 ± 0.16 capillaries/μm^2^, *p* < 0.05) (Fig. 2d). The size of arterioles was reduced in the diabetic group (Fig. 2e). However, we did not observe any difference in the localisation or density of CD146^+^ VWF^−^ pericytes between the two groups.

### Flow cytometry quantification of pericytes within freshly collected total bone marrow mononuclear cells

**Fig. 3:**
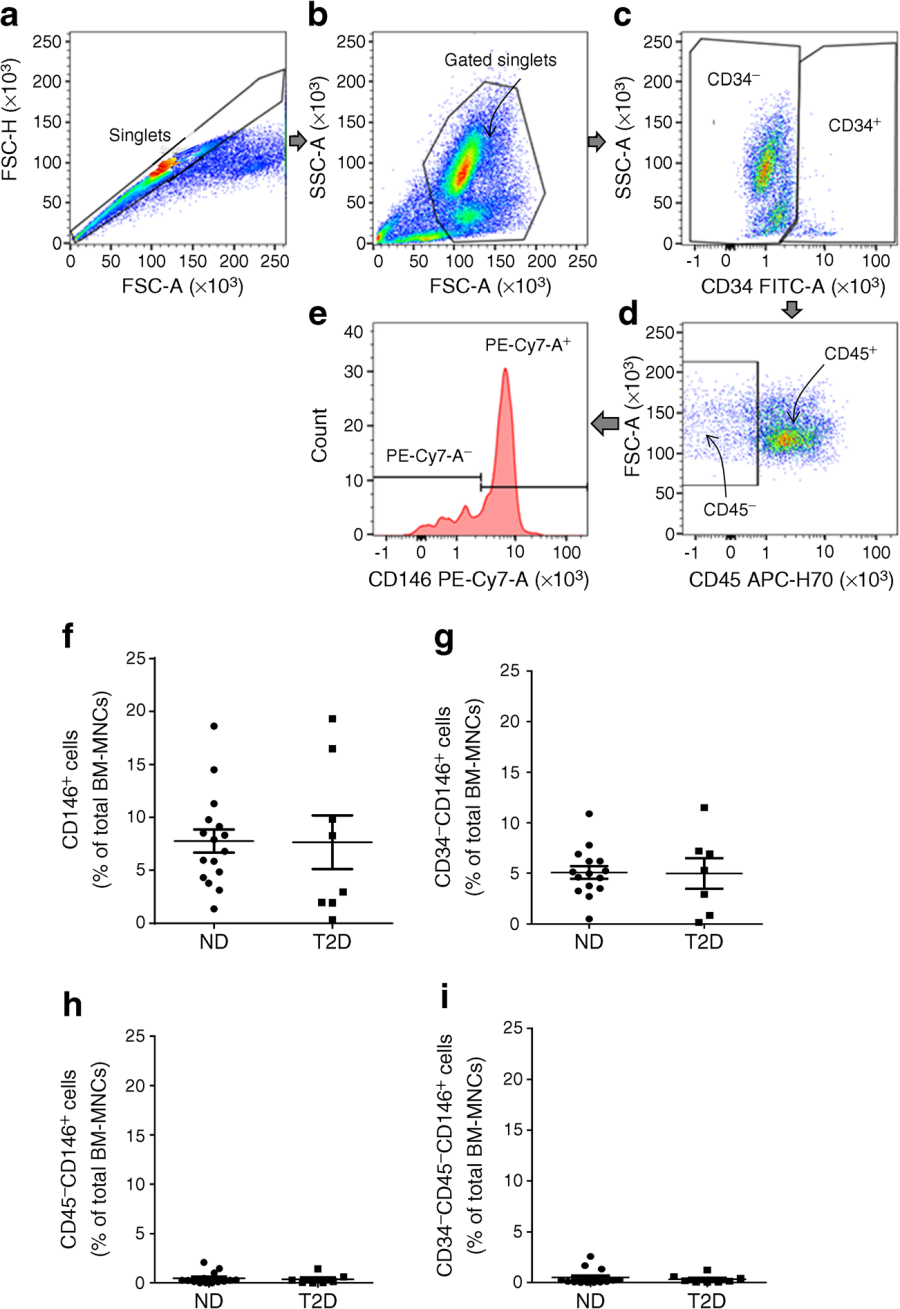
Flow cytometry assessment of bone marrow pericytes. Freshly isolated bone marrow mononuclear cells (BM-MNCs) were analysed by flow cytometry for CD146, CD34 and CD45 markers. (**a**–**e**) The gating strategy consisted of selecting singlet populations using FSC-height (FSC-H) by FSC-area (FSC-A) (**a**), followed SSC-A by FSC-A (**b**) to exclude false-positive events, which are outside the indicated boundaries. Following this, the total BM-MNC population was gated according to the expression of the surface antigen CD34 (**c**). CD34^−^ events were gated and further analysed for CD45 (**d**). Finally, the CD45^−^CD34^−^ cell population was assessed according to CD146 positivity, using the fluorophore PE-Cy7-A (**e**). (**f**–**i**) Bar graphs showing the abundance of CD146^+^ (**f**), CD34^−^CD146^+^ (**g**), CD45^−^CD146^+^ (**h**) and CD34^−^CD45^−^CD146^+^ cells (**i**) within BM-MNCs from non-diabetic (*n*=15) and diabetic (*n*=10) individuals. Data are expressed as individual values and mean±SEM. Analysis was performed using Student’s *t* test. FSC, forward scatter; ND, non-diabetic; SSC, side scatter; T2D, type 2 diabetes

We next performed flow cytometry analysis of the abundance of pericytes within bone marrow mononuclear cells. Fig. 3a–e shows the gating strategy and Fig. 3f–i illustrates the individual values from different bone marrow samples for the rate of CD146^+^ (Fig. 3f), CD34^−^CD146^+^ (Fig. 3g), CD45^−^CD146^+^ (Fig. 3h) and CD34^−^CD45^−^CD146^+^ cells (Fig. 3i), the latter combination being previously used to identify human bone marrow pericytes [33]. There was no difference in the number of CD34^−^CD45^−^CD146^+^ pericytes from non-diabetic vs diabetic individuals: CD34^−^CD45^−^CD146^+^ pericytes accounted for 0.50 ± 0.19% (non-diabetic) and 0.33 ± 0.15% (diabetic) of total BM-MNCs (Fig. 3i).

### Flow cytometry antigenic characterisation of expanded bone marrow CD146^+^ pericytes

**Fig. 4:**
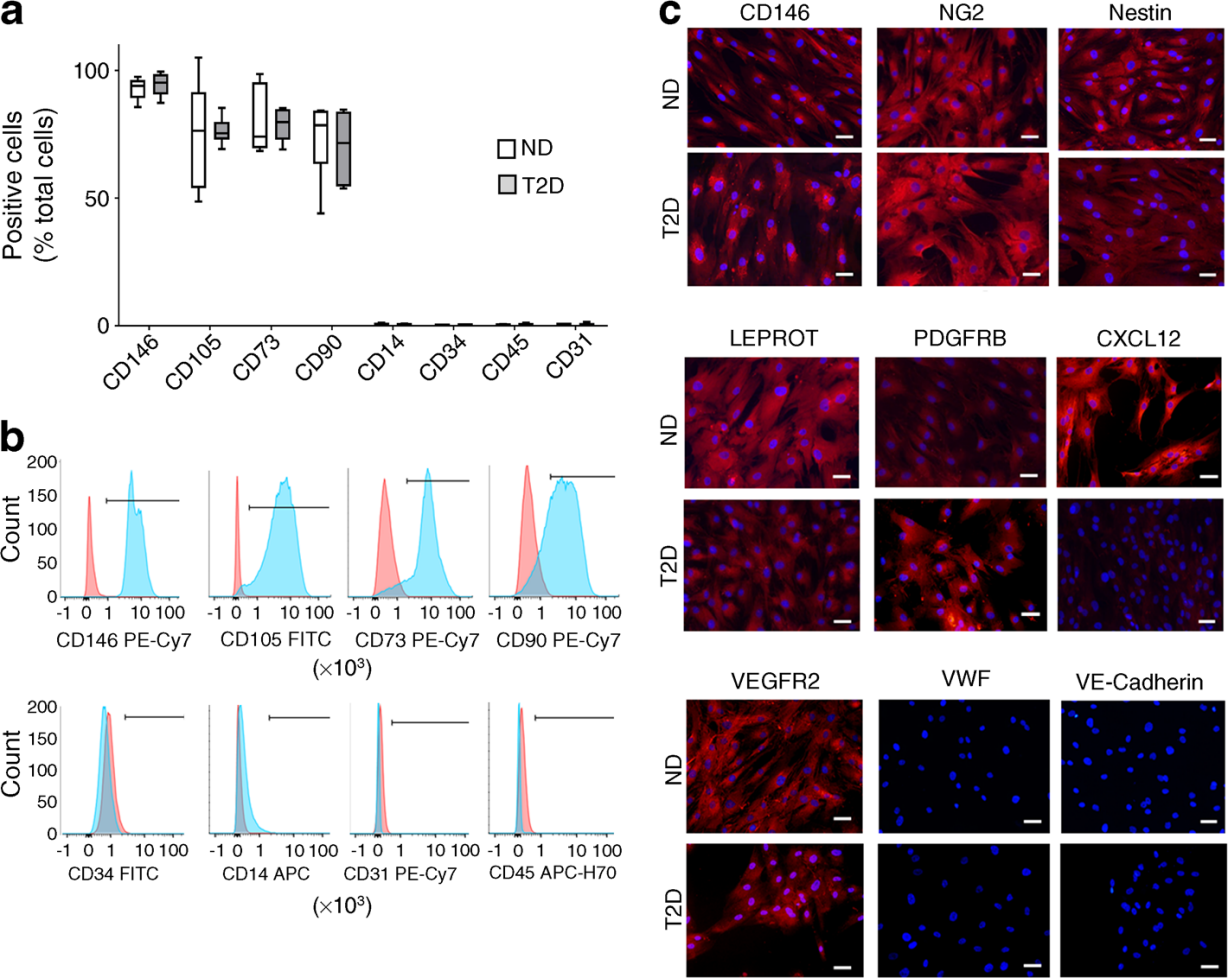
Characterisation of CD146^+^ pericytes following culture expansion. CD146^+^ pericytes were selected through immunomagnetic sorting and then expanded. (**a**, **b**) Flow cytometry data showing a similar profile of expanded cells in non-diabetic and diabetic individuals. Cells expressed CD146, CD105, CD73 and CD90 and were negative for CD14, CD34, CD45 and CD31. *n*=6 per group. The bar and whisker graph shows mean, 25th–75th percentile and minimum and maximum values (**a**). Analysis was performed by Student’s *t* test. (**b**) Histogram overlays with positive staining illustrated by the light blue shading and isotype control by the pink shading. (**c**) Fluorescent microscopy immunostaining images showing cell positivity for CD146, NG2, nestin, LEPROT, PDGFRβ, CXCL12 and VEGFR2. Expanded CD146^+^ pericytes did not express VWF or VE-cadherin. Scale bars, 50 μm. ND, non-diabetic; T2D, type 2 diabetes

We next assessed the antigenic profile of CD146^+^ immunosorted bone marrow pericytes after expansion in culture. Flow cytometry analyses confirmed the maintenance of CD146 abundance in cells from non-diabetic (92.8 ± 1.8%) and diabetic individuals (94.5 ± 1.9%) (Fig. 4a,b). The expanded cells were also strongly positive for mesenchymal markers, such as CD105 (non-diabetic, 74.0 ± 8.1%; diabetic, 76.3 ± 2.2%), CD73 (non-diabetic, 79.9 ± 5.3%; diabetic, 78.8 ± 2.8%) and CD44 (non-diabetic, 74.6 ± 5.0%; diabetic, 70.0 ± 5.5%). Both groups lacked expression of haematopoietic and endothelial markers. Fluorescent immunocytochemistry showed that bone marrow pericytes expressed NG2 as well as nestin, LEPROT, PDGFRβ and VEGFR2 but were negative for VWF and VE-cadherin (Fig. 4c). The CXCL12 signal was almost absent in CD146^+^ pericytes from diabetic individuals. The reduction in CXCL12 expression was confirmed by qPCR and ELISA (see below).

### Diabetes causes dysfunction of bone marrow pericytes

**Fig. 5:**
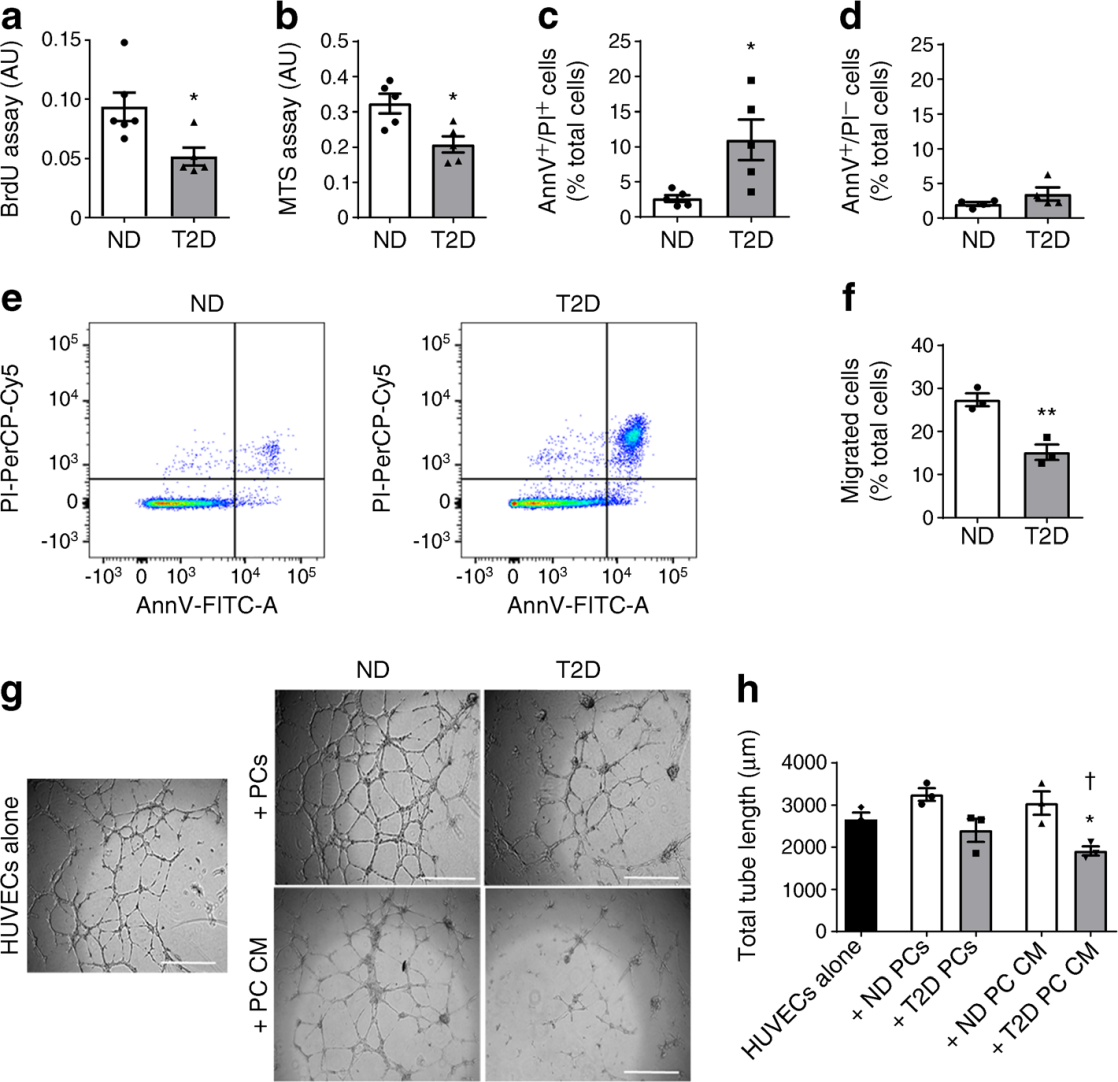
Diabetes alters the functional profile of CD146^+^ pericytes. Bar graphs showing the results of BrdU assay (**a**), MTS assay (**b**) and flow cytometry analysis of early (**c**) and late apoptosis (**d**). (**e**) Representative plots of flow cytometry data shown in (**c**) and (**d**). (**f**) Bar graph showing the results of the migration assay in which CD146^+^ pericytes were exposed to a gradient of PDGFB in a transwell chemotactic assay. (**g**, **h**) Representative images (**g**) and bar graph (**h**) of the Matrigel assay, showing a reduced ability of HUVECs to form capillary-like structures upon co-culture with CD146^+^ pericytes from individuals with type 2 diabetes or their conditioned medium. *n*=5 (**a**–**d**) or *n*=3 per group (**f**, **h**). Data are shown as individual values and mean ± SEM; *p<0.05, **p<0.01 vs non-diabetic group; ^†^*p*<0.05 vs HUVECs alone, Student’s *t* test. AnnV, Annexin V; CM, conditioned medium; ND, non-diabetic; PC, pericytes; PI, propidium iodide; T2D, type 2 diabetes

We next investigated the impact of diabetes on pericytes’ function. Both viability and proliferation were significantly reduced in pericytes from diabetic individuals (*p* < 0.05 for both comparisons, Fig. 5a,b), which also showed a fourfold increase in the abundance of late apoptotic (Annexin V^+^/propidium iodide^+^) events (*p* < 0.05). The fraction of early apoptotic cells did not differ between groups (Fig. 5c–e).

Pericytes from diabetic individuals showed an impaired migration capacity towards a PDGFB gradient (Fig. 5f). Pericytes from non-diabetic individuals were able to support the formation of networks by HUVECs either directly in a co-culture system or indirectly through factors contained in their conditioned medium (*p* < 0.05 vs HUVECs alone) (Fig. 5g showing representative images and Fig. 5h showing mean and individual values). These effects were nullified by diabetes. Moreover, the conditioned medium of diabetic pericytes exerted an inhibitory effect on HUVEC network formation (*p* < 0.05 vs HUVECs alone and *p* < 0.05 vs conditioned medium of control pericytes) (Fig. 5g showing representative images and Fig. 5h showing mean and individual values). All functional assays were performed under ‘normal glucose’ conditions, therefore the observed alterations are compatible with persistent metabolic memory of the diabetic condition.

### Diabetes alters the angiocrine activity of bone marrow pericytes

**Fig. 6:**
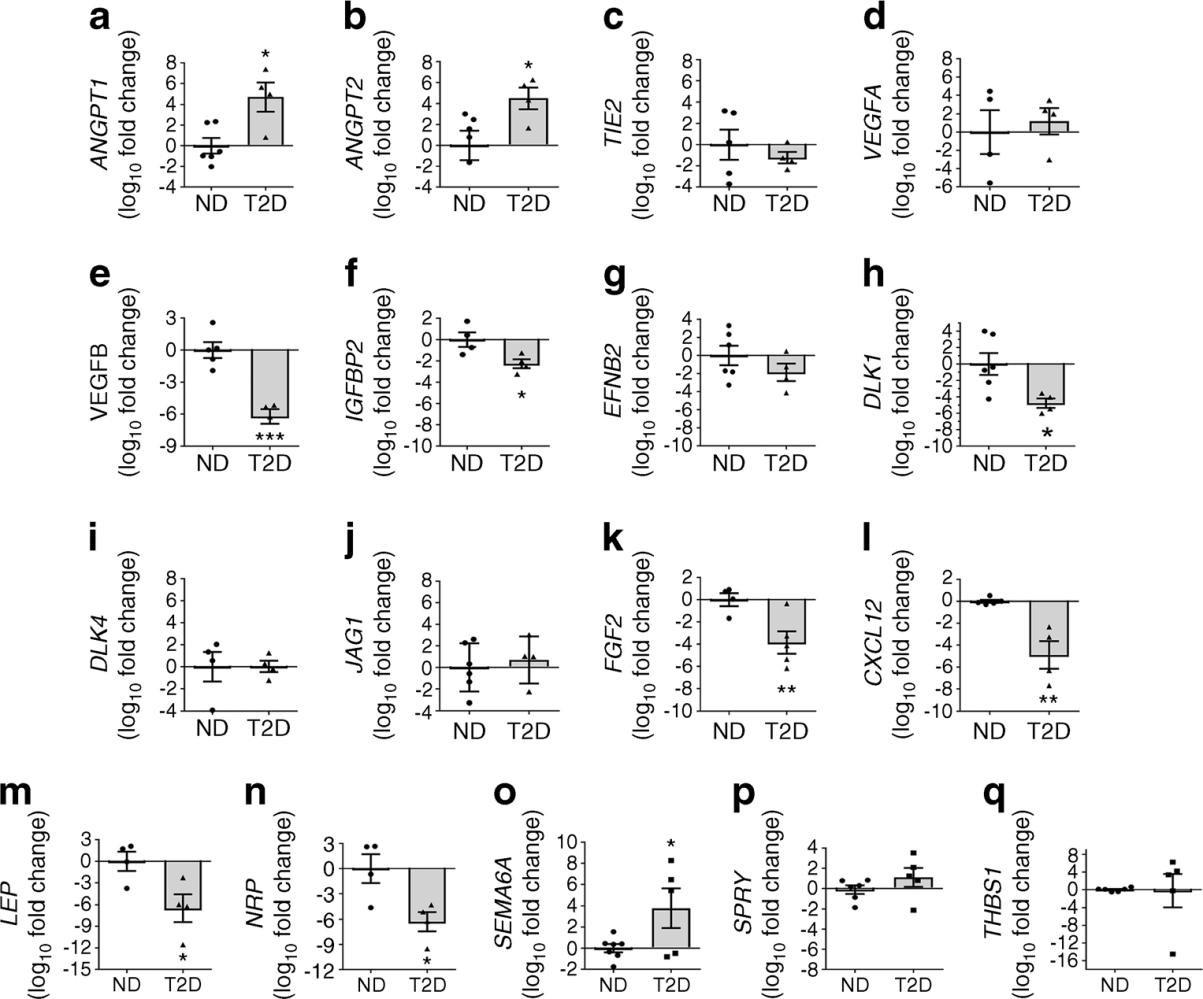
Diabetes alters the gene expression profile of CD146^+^ pericytes. Bar graphs showing results of qPCR analysis performed for *ANGPT1* (**a**), *ANGPT2* (**b**), *TIE2* (**c**), *VEGFA* (**d**), *VEGFB* (**e**), *IGFBP2* (**f**), *EFNB2* (**g**), *DLK1* (**h**), *DLK4* (also known as *DLL4*) (**i**), *JAG1* (**j**), *FGF2* (**k**), *CXCL12* (**l**), *LEP* (**m**), *NRP* (**n**), *SEMA6A* (**o**), *SPRY* (**p**) and *THBS1* (**q**). Data are expressed as individual values and mean ± SEM, fold change vs non-diabetic group; *n*=6 (non-diabetic group) or *n*=4 (diabetic group). *p<0.05, **p<0.01, ***p<0.001 vs non-diabetic group, Student’s *t* test. ND, non-diabetic; T2D, type 2 diabetes

We next investigated the mRNA levels of angiocrine factors in pericytes and corresponding protein expression in the conditioned medium. Both *ANGPT1* and *ANGPT2* mRNA expression were upregulated in bone marrow pericytes from diabetic individuals (*p* < 0.05 both comparisons), whereas *TIE2* (also known as *TEK*), the gene encoding the ANGPT receptor (tyrosine kinase with immunoglobulin and epidermal growth factor homology domain-2 [TIE2]), remained unchanged (Fig. 6a–c). *VEGFA* was not affected by diabetes, whereas *VEGFB* was significantly downregulated (*p* < 0.001 vs the control group, Fig. 6d,e). This was associated with a decrease in the gene encoding insulin-like growth factor-binding protein 2 (*IGFBP2*) (*p* < 0.05 for diabetic vs non-diabetic individuals, Fig. 6f), a transcriptional activator of VEGF [35]. In contrast, the ephrin B2 gene (*EFNB2*), which reportedly controls VEGFR2 internalisation and signalling [36], was expressed at a similar level in the two groups (Fig. 6g). NOTCH signalling in endothelial cells and pericytes acts downstream of VEGF to shape the vascular network during angiogenesis [37]. We found that the gene encoding Notch ligand delta-like 1 (*DLK1*) was downregulated by diabetes (*p* < 0.05 vs non-diabetic individuals), whereas the genes encoding delta-like 4 (*DLK4*) and jagged 1 (*JAG1*) remained unchanged (Fig. 6h–j). A trio of genes encoding angiogenic factors—*FGF2*, *CXCL12* and *LEP*—were downregulated by diabetes (*FGF2* an *CXCL12*, *p* < 0.01 vs control group; *LEP*, *p* < 0.05 vs control group; Fig. 6k–m). Neuropilins play a central role in blood vessel physiology as receptors for semaphorin (SEMA) and VEGF isoforms [38, 39]. Here, we found diabetes reduced *NRP1* transcripts in bone marrow pericytes and increased *SEMA6A* (*p* < 0.05 vs non-diabetic control individuals for both comparisons, Fig. 6n,o), a known angiogenesis inhibitor [40]. Diabetes did not alter genes encoding the two angiogenesis repressors sprouty (*SPRY*) [41] and thrombospondin (*THBS1*) (Fig. 6p,q) [42].

**Fig. 7:**
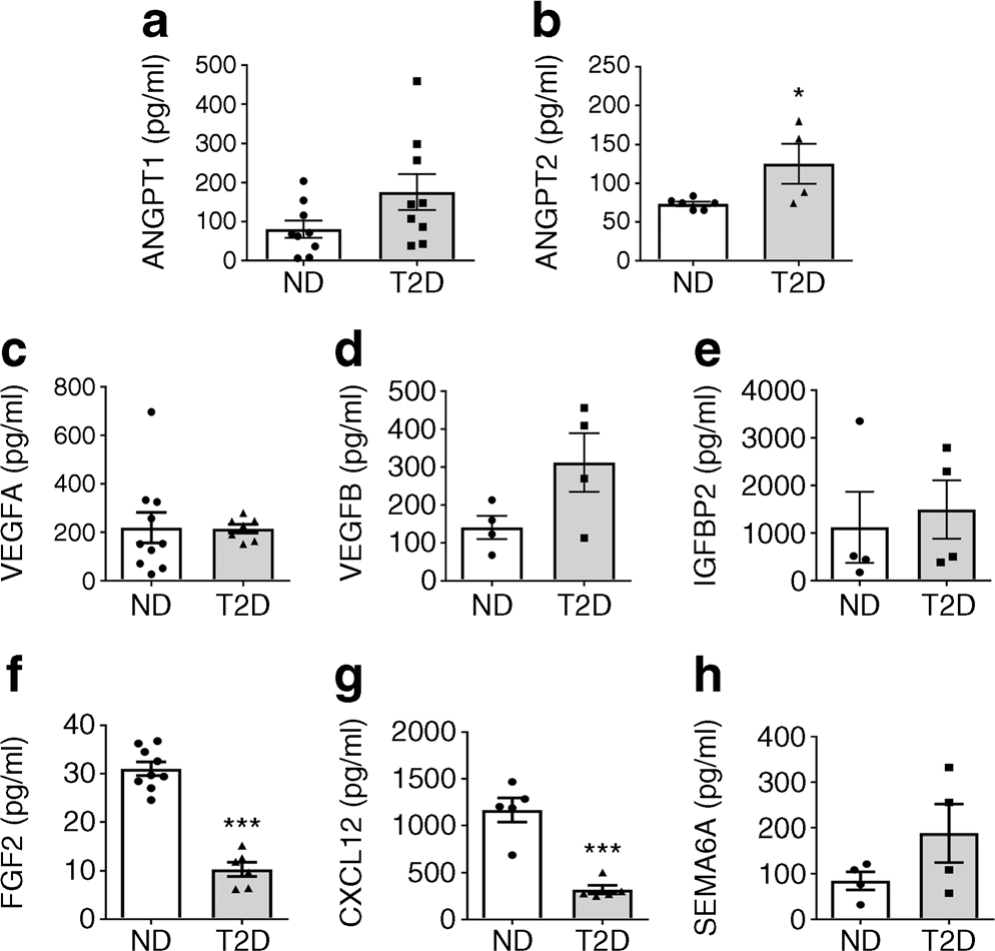
Diabetes alters the secretome of CD146^+^ pericytes. Bar graphs showing results of ELISA for ANGPT1 (**a**), ANGPT2 (**b**), VEGFA (**c**), VEGFB (**d**), IGFBP2 (**e**), FGF2 (**f**), CXCL12 (**g**) and SEMA6A (**h**). Data are shown as individual values and mean±SEM; *n*=4–10 (non-diabetic group) and *n*=4–9 (diabetic group). *p<0.05, ***p<0.001 vs non-diabetic group, Student’s *t* test. ND, non-diabetic; T2D, type 2 diabetes

We next performed ELISA of secreted factors in the pericyte conditioned medium (Fig. 7). Diabetes induced an increase in immunoreactive ANGPT2 (*p* < 0.05) but did not alter ANGPT1, VEGFA, VEGFB, IGFBP2 or SEMA6A (Fig. 7a–e,h). Moreover, FGF2 and CXCL12 were reduced by diabetes at the protein level (*p* < 0.001, Fig. 7f,g).

### AKT forced expression and ANGPT2 inhibition rescue diabetes-induced pericyte dysfunction

AKT activity controls the ability of bone marrow endothelial cells to secrete angiocrine factors [43, 44] and AKT suppresses ANGPT2 secretion [45]. Moreover, type 1 diabetes inhibits AKT activity in bone marrow endothelial cells [15, 16]. Here, we found that type 2 diabetes does not alter AKT phosphorylation in bone marrow pericytes (see electronic supplementary material [ESM] Fig. 1). Nonetheless, AKT might be uncoupled from downstream signalling. Therefore, we attempted to rescue the pericytes’ function by either transducing them with constitutively active myristoylated *AKT* or inhibiting ANGPT2 by adding a blocking antibody to the conditioned medium.

**Fig. 8:**
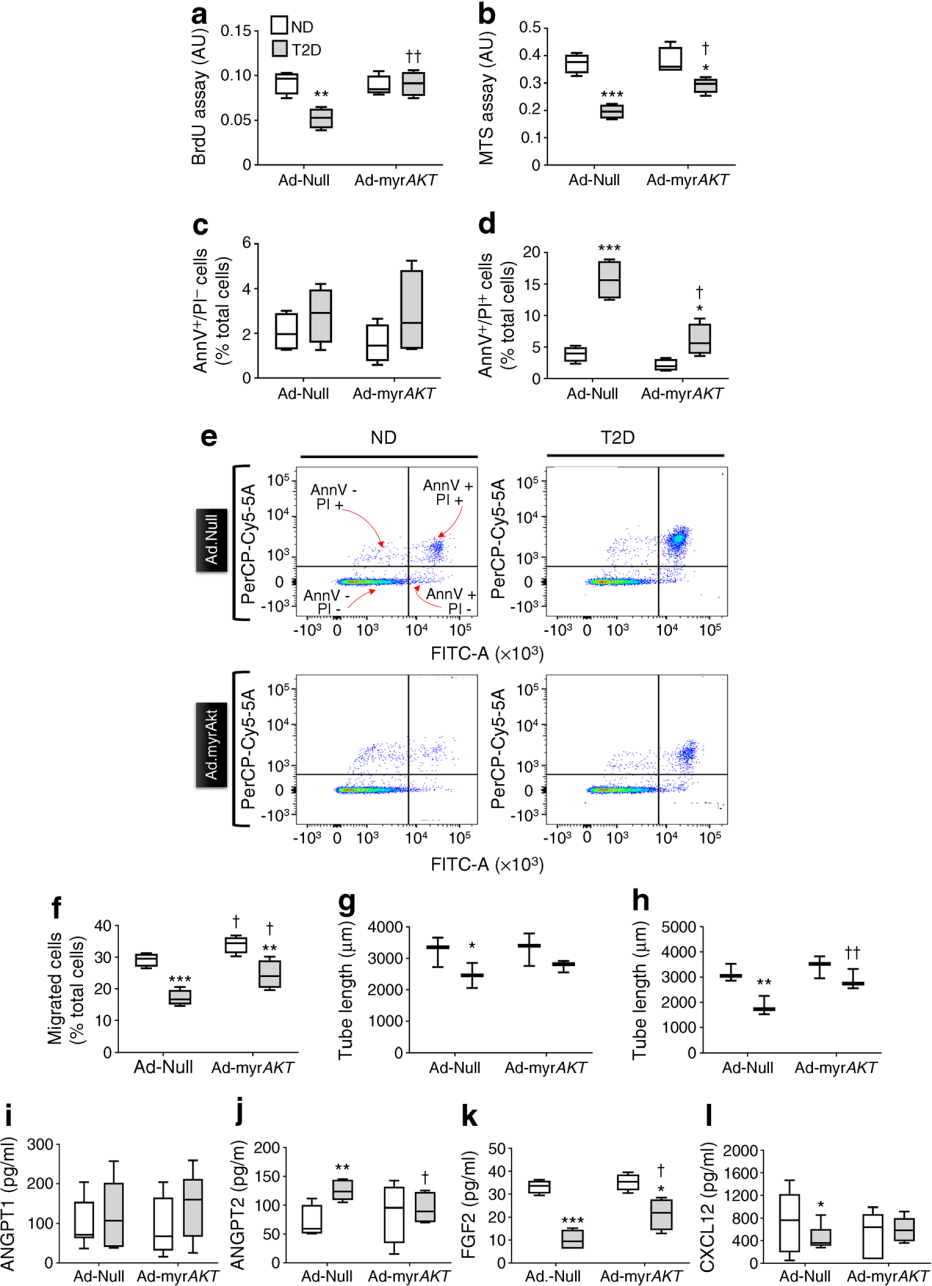
The presence of Ad-myr*AKT* rescues the dysfunction of CD146^+^ pericytes taken from diabetic individuals. CD146^+^ pericytes from non-diabetic or diabetic individuals were infected with Ad-myr*AKT* or Ad-Null and were then assessed for proliferation (**a**), viability (**b**), apoptosis (**c**–**e**) and migration (**f**). The gating strategy to distinguish different subfractions of cells based on the expression of Annexin V and PI is shown in the representative scattergrams (**e**). CD146^+^ pericytes from non-diabetic or diabetic individuals were also assessed for capacity to support angiogenesis in a Matrigel assay with HUVECs, either in co-culture (**g**) or using the CD146^+^ pericyte conditioned medium (**h**). (**i–l**) Graphs showing the results of ELISA performed on the conditioned media of CD146^+^ pericytes from non-diabetic or diabetic individuals infected with Ad-myr*AKT* or Ad-Null; ANGPT1 (**i**), ANGPT2 (**j**), FGF2 (**k**) and CXCL12 (**l**) were measured. Bar and whisker graphs show mean, 25th–75th percentile and minimum and maximum values; *n*=5 per group. *p<0.05, **p<0.01, ***p<0.001 vs the corresponding Ad-myr*AKT* or Ad-Null infected non-diabetic group; ^†^*p*<0.05 and ^††^*p*<0.01 vs Ad-Null infected diabetic group, ANOVA followed by Bonferroni post hoc *t* test. The key in (**a**) applies to all figure parts. AnnV, Annexin V; ND, non-diabetic; PerCP, peridinin chlorophyll protein complex; PI, propidium iodide; T2D, type 2 diabetes

The presence of Ad-myr*AKT* did not affect the proliferation, viability or apoptosis of pericytes from non-diabetic individuals. In contrast, in pericytes from diabetic individuals, infection with Ad-myr*AKT* resulted in increased proliferation (*p* < 0.01 vs Ad-Null, Fig. 8a), improved pericyte viability (*p* < 0.01, Fig. 8b) and reduced late apoptosis (*p* < 0.05, Fig. 8c–e). In pericytes from the non-diabetic group, Ad-myr*AKT* increased the migratory activity of pericytes towards PDGFB (*p* < 0.05 vs Ad-Null, Fig. 8f) but did not enhance their angiogenic activity (Fig. 8g,h). In pericytes from diabetic individuals, Ad-myr*AKT* blunted the migratory deficit (*p* < 0.05 vs Ad-Null) but did not abolished the difference vs non-diabetic group (*p* < 0.01, Fig. 8f). Ad-myr*AKT* abrogated the detrimental effect of diabetes on the promotion of endothelial networking by pericytes or their conditioned medium (*p* < 0.01 vs Ad-Null, Fig. 8g,h). This effect was associated with a reduction in ANGPT2 (*p* < 0.05) and an increase in FGF2 levels (*p* < 0.01) (Fig. 8i–l).

**Fig. 9:**
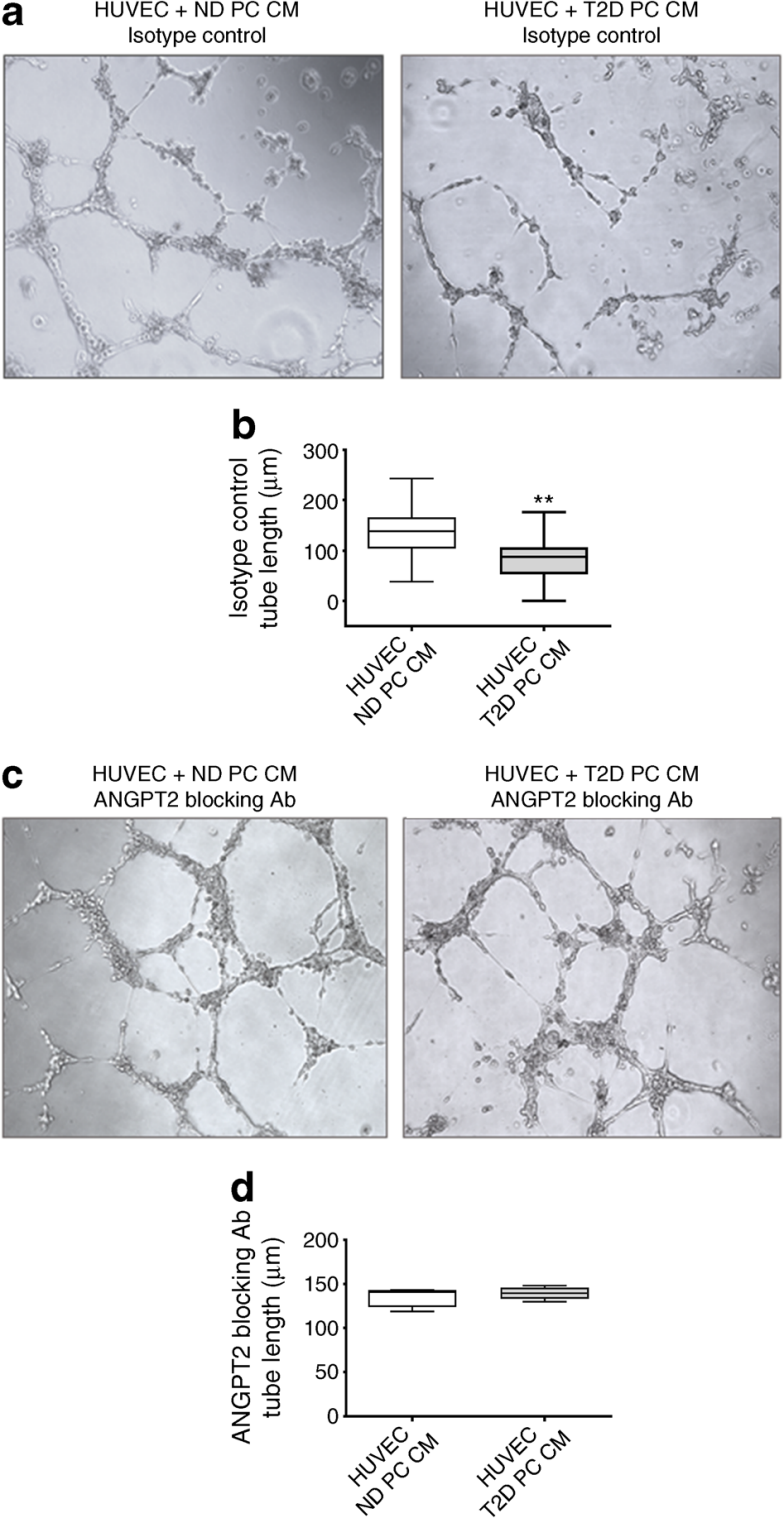
Inhibition of ANGPT2 by a blocking antibody restores the ability of pericytes from diabetic individuals to promote endothelial network formation. Pericytes from non-diabetic or diabetic individuals were treated with anti-ANGPT2 antibody or with IgG isotype control. Their conditioned media was harvested and used to stimulate the formation of networks by HUVECs on Matrigel. (**a**, **b**) Representative images (**a**) and bar graph (**b**) showing that conditioned media of pericytes from diabetic individuals inhibits endothelial network formation compared with conditioned media of pericytes from non-diabetic individuals. (**c**, **d**) Representative images (**c**) and bar graph (**d**) showing that the inhibition exerted by the conditioned media of pericytes from diabetic individuals is abrogated by an ANGPT2 blocking antibody. Bar and whisker graphs show mean, 25th–75th percentile and minimum and maximum values. *n*=3 per group. **p<0.01 vs non-diabetic, Student’s *t* test. Ab, antibody; CM, conditioned medium; ND, non-diabetic; PC, pericyte; T2D, type 2 diabetes

Addition of an ANGPT2 blocking antibody to the pericyte conditioned media abrogated the inhibitory effect of diabetes on promotion of endothelial network formation (*p* < 0.01 vs IgG isotype control, Fig. 9a–d), suggesting that excess production of ANGPT2 may be accountable for the loss of angiogenic activity of bone marrow pericytes in diabetes.

## Discussion

Pericytes provide support to the vascular endothelium through direct contacts and paracrine signalling [46]. Furthermore, stem cell properties [47–50] and reparative capacities [51–55] have been attributed to pericytes. Under pathological conditions, such as diabetes [56] and ischaemia [57], dysfunctional pericytes may participate in organ damage. The present study shows several deficits accrue in bone marrow pericytes from individuals with type 2 diabetes.

In 2007, Sacchetti et al were the first to demonstrate the existence of clonogenic CD146^+^ cells in the bone marrow of individuals with fibrous dysplasia [27]. These cells express a host of pericyte markers, such as αSMA, NG2, PDGFRβ and calponin 1 and 3, secrete JAG1, CXCL12 and ANGPT1 and are capable of transferring, upon transplantation, the haematopoietic microenvironment to heterotopic sites. Subsequently, Corselli et al showed that clonogenically expanded CD34^−^CD45^−^CD146^+^ pericytes from human fetal bone marrow are capable of establishing multilineage haematopoiesis in immunodeficient mice. In contrast, unsorted mesenchymal cells or CD146^−^ cells promoted rapid HSPC differentiation with consequent loss of engraftment ability [28]. These studies established the concept that bone marrow CD146^+^ cells belong to the class of microvascular pericytes, represent the human counterpart of murine bone marrow Cxcl12-abundant reticular cells or nestin^+^ cells, and play key roles in the maintenance and functionality of the vascular niche, supporting endothelial cell stability and haematopoiesis.

The present investigation compared the abundance, localisation and characteristics of bone marrow CD34^−^CD45^−^CD146^+^ pericytes in a cohort of 25 non-diabetic individuals and 14 individuals with uncomplicated type 2 diabetes referring to our Orthopaedic Center for hip reconstructive surgery. We found that bone marrow CD146^+^ pericytes typically localise around capillaries and sinusoids, with no evidence of different abundance between samples from diabetic and non-diabetic individuals, as assessed by in situ immunohistochemistry and flow cytometry. CD34^−^CD45^−^CD146^+^ pericytes accounted for 0.50% (non-diabetic) and 0.33% (diabetic) of total BM-MNCs. These figures are higher than those reported by Sacchetti et al, who found these cells represent 0.11 ± 0.02% of total BM-MNCs from bone marrow of individuals with fibrodysplasia, a disease characterised by ossification of connective tissue [27].

Furthermore, we confirmed that immunosorted CD34^−^CD45^−^CD146^+^ pericytes could be expanded in culture, where they maintain the expression of typical markers, such as CD105, CD73, CD90, NG2, nestin and PDGFRβ [27, 28]. Importantly, we discovered that pericytes expanded from the bone marrow of diabetic individuals have functional alterations, such as reduced proliferation, viability and capacity to migrate towards a chemoattractant stimulus and support endothelial networking. These alterations were associated with a distinctive molecular signature. For instance, CXCL12 was downregulated at mRNA and protein level. Previous studies have highlighted the secretion of CXCL12 by bone marrow stromal cells as representing a pivotal mechanism essential not only for the steady-state egress and rapid mobilisation of HSPCs into the circulation but also for the maintenance of a proper endothelial cell barrier integrity [58, 59]. The decrease of CXCL12 in diabetic pericytes is therefore in keeping with an altered function of the bone marrow vascular niche [4, 60].

In addition, the cellular mRNA levels of several angiocrine factors were altered by diabetes. These included *ANGPT1*, *ANGPT2* and *SEMA6A* (upregulated) and *VEGFB*, *IGFBP2*, *DLK1*, *FGF2*, *LEP* and *NRP1* (downregulated). We validated seven of the nine differentially regulated genes by measuring their protein levels in the pericyte conditioned medium and found a concordance between transcriptional and post-transcriptional data for FGF2 and ANGPT2. FGF2 exerts proliferative effects on both pericytes and endothelial cells through activation of mitogen-activated protein kinases (MAPK) and AKT, whereas its removal causes withdrawal from the growth cycle, inducing pericytes to acquire a smooth-muscle-like contractile phenotype [61]. Moreover, FGF2 reportedly promotes self-renewal of HSPCs [62].

Stromal cell-produced ANGPT1 plays a pivotal role in the maintenance of haematopoietic stemness [63]. Moreover, *CD146* or *ANGPT1* gene knockout in bone marrow pericytes reportedly interferes with the pericytes’ ability to direct the remodelling of pseudovascular structures, thus suggesting that ANGPT1 is key for the maintenance of the proper interaction between pericytes and endothelial cells [33]. In contrast, the roles of ANGPT2 within the bone marrow vascular niche remain unknown. Until recently, the vascular ANGPT–TIE2 signalling has been exclusively related to TIE2 expression by endothelial cells [64]. ANGPT1 secreted by pericytes binds to TIE2 receptors on endothelial cells, thus supporting cell survival rate, vessel stability and endothelial barrier function through the serine kinase AKT–forkhead box protein O1 signalling pathway [65]. ANGPT2 is mainly expressed and released by endothelial cells and acts as a context-dependent weak TIE2 agonist or antagonist and a destabiliser of endothelial integrity [66]. A recent study showed that TIE2 receptors are also expressed by pericytes and participate in the ANGPT-dependent crosstalk with endothelial cells [67].

Our results further integrate the above scheme. In fact, while confirming human bone marrow pericytes express ANGPT1 and TIE2, we also show these cells express and release ANGPT2. These new data suggest the presence of an autonomous ANGPT2 signalling in pericytes, integrating the paracrine ANGPT2 signalling from endothelial cells. Importantly, we found that bone marrow pericytes from diabetic individuals produce excess ANGPT2, which may compete with ANGPT1 for binding to TIE2 on pericytes and endothelial cells. The imbalance between the opposing ANGPTs may contribute to jeopardising the vascular bone marrow niche at multiple levels.

AKT is co-activated in proliferative vascular cells by proangiogenic factors, like ANGPT1 and FGF2, but it can also reciprocally induce a marked upregulation of a spectrum of angiocrine factors. For instance, activation of AKT in primary endothelial cells induces the expression of FGF2, IGFBP2 and ANGPT1, while it suppresses inhibitory factors such as ANGPT2 and DLK1 [43]. Additionally, selective activation of AKT in the bone marrow endothelial cells of adult mice increased long-term haematopoietic repopulation capacity [43]. We found that AKT phosphorylation is not altered in bone marrow pericytes of diabetic individuals. AKT uncoupling from associated signalling pathways remains a possible explanation for the observed pericytes’ dysfunctions. In line with this, transfection with constitutively active Ad-myr*AKT* abrogated pericyte deficits, restored FGF2 and inhibited ANGPT2. This finding reproduces the functional benefit we observed previously by reactivating AKT in human bone marrow endothelial cells in a mouse model of type 1 diabetes [15].

### Conclusion and future perspectives

This study newly identifies a detrimental effect of type 2 diabetes on human bone marrow pericytes, involving the inactivation of the ANGPT–FGF2 angiocrine signalling pathway. Given the limited group size and the unavailability of recent HbA_1c_ measurements in some of the participants, it was not possible to conclude that pericyte dysfunction correlates with metabolic control. Interestingly, pericytes were expanded and functionally assessed under in vitro conditions of normal glucose, thus suggesting a persistent memory of the diabetic environment. These data could have important translational implications and require further investigation. For instance, pericyte dysfunction might contribute to bone marrow microangiopathy, with an indirect impact on proper mobilisation of stem/progenitor cells into the circulation. Furthermore, a reduced angiocrine signalling from pericytes and endothelial cells might contribute to alterations of the haematopoietic system, including cell-intrinsic alterations of haematopoietic stem cells, leading to a decline in self-renewal, immune system dysregulation, predisposition to myeloid neoplasms and delayed haematopoietic recovery after myelosuppression.

## Electronic supplementary material

ESM (PDF 2746 kb)

## Data Availability

All data are available upon request.

## Abbreviations

Ad-myr*AKT*: Adenoviral vector carrying the coding sequence for constitutively active myristoylated Akt
Ad-Null: Adenoviral empty vector
ANGPT1: Angiopoietin 1
ANGPT2: Angiopoietin 2
BM-MNC: Bone marrow mononuclear cell
CXCL12: Chemokine (C-X-C motif) ligand 12
DLK1: Delta-like 1
DLK4: Delta-like 4
FGF2: Fibroblast growth factor 2
HSPC: Haematopoietic stem/progenitor cell
IGFBP2: Insulin-like growth factor-binding protein 2
JAG1: Jagged 1
KITLG: Stem cell factor–KIT ligand
LEPROT: Leptin receptor
NG2: Neural/glial antigen 2
PDGFB: Platelet-derived growth factor B
PDGFRα: Platelet-derived growth factor receptor α
PDGFRβ: Platelet-derived growth factor receptor β
PGP9.5: Protein gene product 9.5
qPCR: Quantitative PCR
SEMA: Semaphorin
αSMA: α-Smooth muscle actin protein
TIE2: Tyrosine kinase with immunoglobulin and epidermal growth factor homology domain-2
VE-cadherin: Vasclular endothelial-cadherin
VEGF: Vascular endothelial growth factor
VEGFR2: Vascular endothelial growth factor receptor 2
VWF: von Willebrand factor
