# Supplementary material for: Bone marrow pericyte dysfunction in individuals with type 2 diabetes
Source: Diabetologia. 2019 Apr 17;62(7):1275–90. doi: 10.1007/s00125-019-4865-6 (PMC6560025; doi:10.1007/s00125-019-4865-6)
Supplement: Supplementary file 1 — (PDF 2746 kb) [file 125_2019_4865_MOESM1_ESM.pdf]

ESM Table 1 Antibodies used for flow cytometry

Antigen	Fluorophore	Supplier	Cat#	Dilution
CD105	FITC	Thermo Scientific	MA1-19594	1/25
CD14	APC	BioLegend	301808	1/30
CD146	PE-Cy7	BD Biosciences	562135	1/10
CD16	PerCP	BioLegend	302030	1/20
CD184	APC	BD Biosciences	555976	1/10
CD31	PE	BD Biosciences	560983	1/10
CD34	FITC	Miltenyi	130-081-001	1/20
CD44	PE	BD Biosciences	550989	1/10
CD45	APC-H7	BD Biosciences	560178	1/10
CD73	PE-Cy7	BD Biosciences	344010	1/10
CD90	PE	BD Biosciences	555596	1/10

ESM Table 2 List of antibodies used for western blot analyses

Protein target	Supplier	Catalogue number
p-AKT (Ser473)	Cell Signalling Technology	9271
p-AKT (Thr308)	Cell Signalling Technology	9275
AKT	Cell Signalling Technology	9272
β-Tubulin	Cell Signalling Technology	86298
β-Actin	Sigma-Aldrich	A5441 (AC15)

ESM Table 3 List of antibodies used for immunohistochemistry (IHC) and immunocytochemistry (ICC)

Antigen	Application	Isotype	Clone	Concentration	Brand	Code	Secondary Ab (Alexa Fluor)
Human MCAM	IHC	Mouse IgG	1/MCAM	1/ 50	BD Transduction Laboratories	611208	Mouse dev. Goat 568
Von Willebrand Factor	IHC	Rabbit IgG	polyclonal	1/ 30	Abcam	ab9378	Rabbit dev. Goat 488
CD146	IHC, ICC	Rabbit IgG	EPR3208	1/ 50	Abcam	ab75769	Rabbit dev. Goat 568
PGP9.5	IHC	Mouse IgG	PGP9.5	1/ 100	Abcam	ab72911	Mouse dev. Goat 488
Human Actin (Smooth Muscle)	IHC	Mouse IgG	1A4	1/ 100	Agilent DAKO	GA611	Mouse dev. Goat 488
Nestin	IHC, ICC	Mouse IgG	GT623	1/ 50	Abcam	ab184543	Mouse dev. Donkey 467
SDF1/CXCL12	IHC, ICC	Rabbit IgG	polyclonal	1/ 100	Cell Signaling	3740S	Rabbit dev. Donkey 568
VE Cadherin	ICC	Rabbit IgG	polyclonal	1/ 50	Abcam	ab33168	Rabbit dev. Donkey 568
PDGF Receptor β	ICC	Rabbit IgG	28E1	1/ 50	Cell Signaling	3169S	Rabbit dev. Donkey 568
NG2	ICC	Rabbit IgG	HMB45	1/50	Abcam	ab83508	Rabbit dev. Goat 488
VEGFR2	ICC	Rabbit IgG	polyclonal	1/50	Abcam	ab9530	Rabbit dev. Goat 488

ESM Table 4 List of primers used for qPCR

Gene	Forward primer (5′–3′)	Reverse primer (5′–3′)
ACTB	5′-TGGACATCCGCAAAGACCTGTA-3′	5′-GGGCAGTGATCTCCTTCTGCAT-3′
ANGPT1	5′-ACAGGAGGATGGTGGTTTGA-3′	5′-CGCTTCTGACATTGCGCTTT-3′
ANGPT2	5′-TCAGTGGCTAATGAAGCTTGAGA-3′	5′-CCGCTGTTTGGTTCAACAGG-3′
CXCL10	5′-GCCATTCTGATTTGCTGCCT-3′	5′-GCAGGTACAGCGTACAGTTCT-3′
CXCL12	5′-ATTCTCAACACTCCAAACTGTGC-3′	5′-ACTTTAGCTTCGGGTCAATGC-3′
DLK1	5′-GCTTTCTAATCGGCTCGGGA-3′	5′-CGGCGACTTTCGTTTTCCTC-3′
DLK4[a]	5’-TTGCCACCAGATGCACTCAT-3’	5′-CACATAGTGGCCGAAGTGGT-3′
EFNB2	5′-CCATGGTAACCAGCCACAGT-3′	5′-CCCCTCTCCCCCATCCTAAA-3′
FGF2	5′-TCCACCTATAATTGGTCAA-3′	5′-CATCAGTTACCAGCTCCCCC-3′
IGFBP2	5′-CGAGGGCACTTGTGAGAAGC-3′	5′-CCAGCTCCTTTCATACCCGAC-3′
JAG1	5′-CGTCATTGTGTTACCTGCGG-3′	5′-TGCGCAGCCTTTTATTCCCT-3′
LEP	5′-AATGCATTGGGGAACCCTGT-3′	5′-AGGAGACTGACTGCGTGTGT-3′
NRP1	5′-GAAACTCCTCTGTCTCCCGC-3′	5′-TGTTTCTGGACCCGTTGGAG-3′
SEMA6A	5′-GCTAGCAGAAAAGGGACTAACG-3′	5′-CGCCTGCTCTCTAGCCAGT-3′

SPRY2	5′-GCGCTTGTAGAAGGGGAGT-3′	5′-ATTCCCCGCTCATGAACA-3′
THBS1	5′-TGACAACAATCAGGATATATGATGA-3′	5′-TTGGGCACATAGGGACAGTT-3′
TIE2	5′-GGGACCCACACTTCCAACAA-3′	5′-TTTGGTATCAGCAGGGCTGG-3′
VEGFA	5′-TGTCTAATGCCCTGGAGCCT-3′	5′-GCTTGTCACATCTGCAAGTAGG-3′
VEGFB	5′-GAGATGTCCCTGGAAGAAC-3′	5′-GAGTGGGATGGGTGATGTCAG-3′
18S	5′-CCCAGTAAGTGCGGGTCATAA-3′	5′-CCGAGGGCCTCACTAAACC-3′
GAPDH	5′-AGCCGCATCTTCTTTTGCGT-3′	5′-TGACGAACATGGGGGCATCA-3′

[a]Also known as *DLL4*

ESM Fig. 1

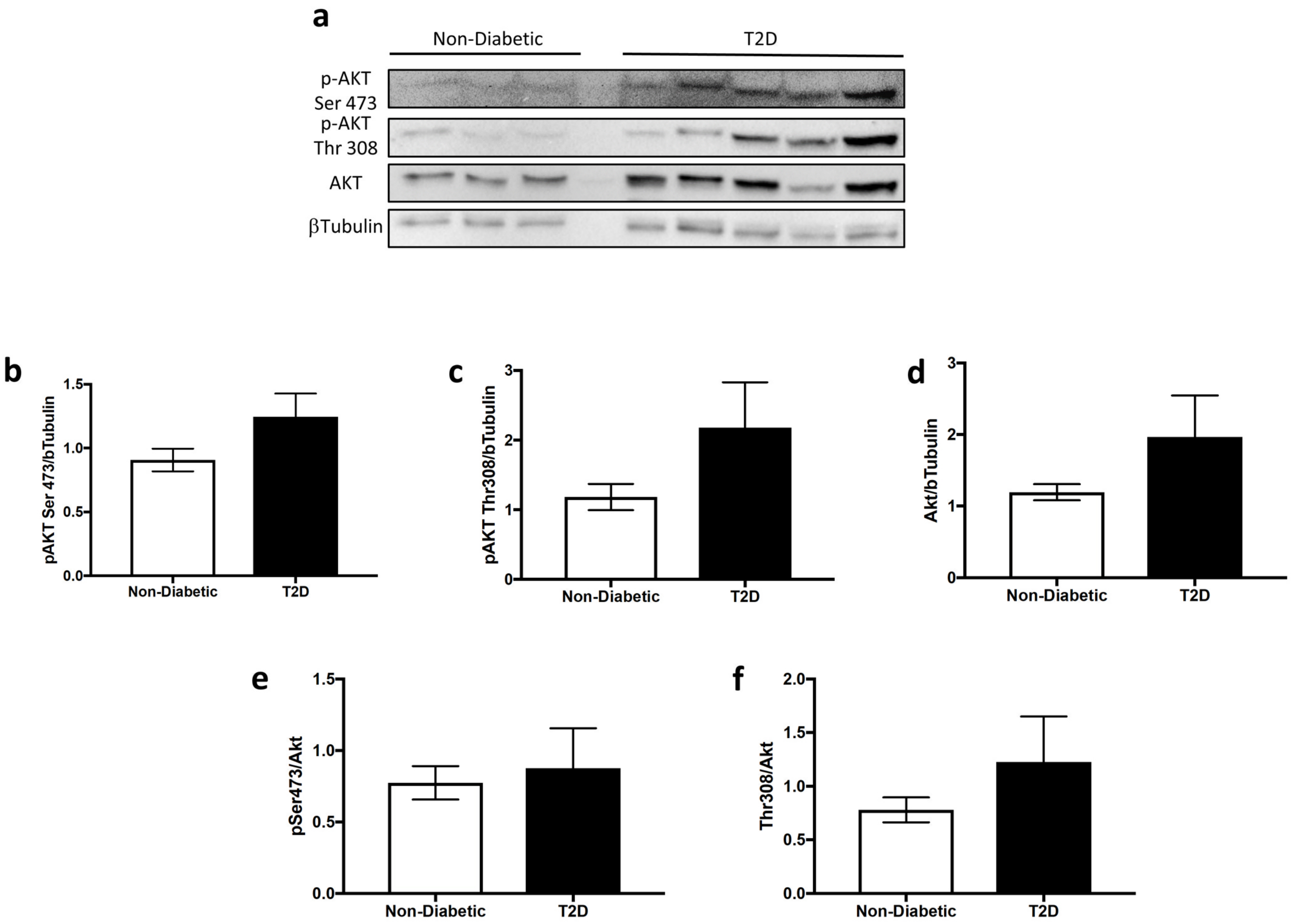

Supplementary Figure 1. Western blot analysis of AKT. (**a**) Blots showing the phosphorylation level of AKT in pericytes isolated from the BM of T2D and nondiabetic patients. Each lane corresponds to an individual cell line. (**b-d**) Bar graphs showing average results expressed as AKT/tubulin ratio. (**e&f**) Bar graphs showing average results expressed as pAKT/total AKT ratio. No significant difference was denoted in the comparison. Analysis was performed by Student's t test.
